# On Spirit of Turpentine in Puerperal Fever

**Published:** 1815-09

**Authors:** Robert Kinglake

**Affiliations:** Taunton.


*For the London Medical and Physical Journal.*

### *On Spirit of Turpentine in Puerperal Fever;* by Dr. Kinglake.

IT is consoling to humanity to learn, by the report of the case given in your last Journal, by Mr. Atkinson, the transcendant efficacy of Spirit of Turpentine, in a late stage of that most untractable disease, Puerperal Fever. The reports of Dr. Brenan on this subject were rendered, indeed, almost incredible, from the surprise that was induced by a statement of curative relief having been obtained by means hitherto held to be totally inconsistent with the end in view. If the reported remedy should become unquestionably established by an adequate stock of such evidence as Mr. Atkinson has recorded in its favour, the pathologist will be compelled to review and correct the theory that would unqualifiedly prohibit stimulant treatment in inflammatory disease. It may be justly suspected that the occasional inefficacy of stimulant remedies in the latter stages of inflammatory disease, arises rather from the insufficiency of the power employed, than from the inadmissibility of its quality. It cannot, indeed, be imagined, that, in the dying circumstances of extreme disease, any thing short of a most powerful agent can uphold the sinking remains of life. To attempt it in the ordinary mode, is no more than the aspect of affording assistance, a sort of *solamen miseris,* creditable indeed to humanity, but not to science, in as far as it is administered with feelings of conscious impotency.

Dr. Brenan deserves well of the medical profession, and has still higher claims on the gratitude of mankind at large, for the proposition which he has made in favour of the salutary efficacy of Spirit of Turpentine, when exhibited in doses far exceeding the established *maximum* of that remedy. It is by thus adventuring beyond the confines of established prejudices and adoptions, that important benefits are often realised. Innumerable ages may roll away in the hackneyed use of an admitted practice, without any amelioration being effected in it, if it be held to be correct, because it has not been impugned, or because the delusive veneration of its antiquity may seem to have for ever consecrated it. Against such unmeaning and deceptious sanctions, the love of truth and of the advancement of science should sternly protest. If a disposition to inquiry in physics, as well as in morals, be not fostered and encouraged, the powers and resources of the human mind must be confined in deplorably narrow limits. The grand objects of intellectual existence would be be contravened and defeated, were not suitable invitation to be given to the promotion of every species of useful improvement. Preconceived notions acquire insuperable obstinacy by long and uninterrupted indulgence, and will ultimately arrogate the right of deciding on the doubtful ground of popular and undisputed adoption. Medicine has greatly suffered by this sort of accredited authority, and can only hope to be relieved from the heavy embarrassments under which both its theories and practice labour, by an enlightened and fearless spirit of investigation, by which alone discoveries can be effected of the nature of that recently made and published by Dr. Brenan, on the remedial powers of Spirit of Turpentine, when employed in adequate doses, in a disease so unmanageable and so generally mortal as that of puerperal fever. ROBERT KINGLAKE.

*Taunton.*

